# A study on the impact of life meaning on occupational identity of nursing students in China:a cross-sectional study

**DOI:** 10.1186/s12912-023-01667-1

**Published:** 2023-12-18

**Authors:** Lingjing Qiu, Qunfang Miao, Yueyan Zhao, Li Zhu, Yanling Wang

**Affiliations:** 1 School of Nursing, Hangzhou Normal University, Hangzhou City, Zhejiang Province China; 2 Department of Clinical Psychology, Affiliated Hospital of Hangzhou Normal University, Hangzhou, 311121 China; 3 Tongxiang Health School, Tongxiang City, Zhejiang Province China

**Keywords:** Nursing students, Professional identity, Life meaning, COVID-19

## Abstract

**Background:**

With the alterations of the Chinese epidemic prevention policy, China experienced a nationwide wave of the Coronavirus disease 2019 (COVID-19) pandemic from December 2022 to January 2023.The impact of the COVID-19 pandemic extends beyond individual perceptions of the meaning of life and attitudes toward life and death; it also affects the professional identity of nursing students.This study explored nursing students’ professional identity and life meaning of affected by the pandemic.

**Methods:**

An online survey was conducted using the Chinese version of the Nursing Student Career Identity Scale and the Chinese version of the Meaning in Life Questionnaire to assess the current status of nursing students’ professional identity and sense of life meaning. Data were also collected on participant gender, education, clinical practice experience, and COVID-19 infection.

**Results:**

The scores for nursing students’ sense of professional identity (61.58 ± 16.16) and sense of life meaning (45.29 ± 12.65) were both at an intermediate level. Compared to the scores before the COVID-19 pandemic, Chinese nursing students’ professional identity scores increased, while their sense of life meaning scores decreased. Moreover, we found a positive correlation between professional identity and sense of life meaning (*p* < 0.001); nursing students exhibited a stronger professional identity when they had a relatively higher sense of life meaning than those with a relatively lower sense of life meaning.

**Conclusion:**

Enhancing nursing students’ sense of meaning in life is crucial for maintaining their professional identity. Attention should be given to life education for nursing students and the development of relevant educational curricula.

## Background

Since the Coronavirus disease 2019 (COVID-19) outbreak in China in 2020, the public health sector has been significantly impacted, particularly nursing workers at the frontline facing the virus. Indeed, these healthcare professionals faced high levels of risk and stress during this period. The threat of the virus and increased workload led to a notable increase in anxiety, depression, and sleep disturbances among nurses [1, 2], adversely affecting their work and personal lives. Research has shown that having a sense of life meaning can protect against negative emotions like depression and contribute to psychological well-being [3]. Importantly, a strong sense of meaning allows nurses to value, appreciate, and cherish life, attend to the spiritual needs of patients, assist them in finding meaning in their own lives, and utilize their personal insights to provide compassionate and comprehensive care. At the same time, it strengthens their own professional identity. Therefore, enhancing nurses’ perception of life meaning promotes their psychological well-being and is crucial in improving their professional identity and the quality of nursing services, helping patients establish life meaning.

Work plays an important role in discovering life’s meaning, and understanding how to discover life’s meaning from work is particularly important. Frankl stated that it does not matter what kind of work a person does, but the attitude and approach to the work [4]. Indeed, a positive, creative, and responsible attitude toward work can give meaning to work. This coincides with the concept of occupational identity.Professional identity [5] refers to the recognition of a socially assigned professional role, the heartfelt acceptance of this profession, and the positive perception and positive evaluation of it. In recent decades, nursing has been defined in China as a profession with low academic standing, low social status, and high workload [6]. Due to the poor public image and self-concept of nursing, the professional identity of Chinese nurses was not high in the past [7]. At the same time, as helpers of “death work”, nurses’ special working environment and the impact of stressful events will make them think more about the meaning of life, and the sense of life meaning of Chinese nurses is mostly moderately high in the past years [8]. A study [9] revealed that during the three years of the fight against the epidemic in China, social media coverage portrayed nurses as respected and self-dedicated heroes, the public image of the nursing profession improved, and the professional identity of nurses was enhanced compared to that before the epidemic [10]. However, it should be borne in mind that nurses are at increased risk of COVID-19 infection compared to the general population, with a mortality rate of 54.2% among Chinese healthcare workers at the beginning of the epidemic [11]. Not only do they face higher levels of infection and mortality threats themselves, but they also increase the risk of infection in their families [12], and these fears and stresses may affect the psychological well-being of nursing staff, further affecting the experience of the meaning of life.

Nursing students, representing the future backbone of the nursing profession, face the challenge of understanding the profound aspects of life and death. They must possess the capacity to effectively cope with the negative impact of illness and death on themselves, their colleagues, and even their patients’ families. Nursing students’ social environment and future career paths necessitate a deeper comprehension of these existential matters. Furthermore, nursing students’ professional identity undeniably influences the nursing workforce’s stability. If nursing students enter the profession with a low sense of professional identity, it may result in low job satisfaction, potentially hastening their departure from the field [13]. In the Chinese education system, few high school students willingly choose nursing as their major after taking the National College Entrance Examination [14]. Instead, many students opt for nursing only after being unsuccessful in securing admission to higher-scoring majors. Moreover, within the Chinese nursing education system, there is limited emphasis on nurturing the meaning of life and developing professional identity among nursing students. The focus primarily remains on acquiring theoretical knowledge and practical skills [15].

Previous studies have shown that nursing students’ professional identity is associated with psychological resilience [16], empathy fatigue [17], and death attitudes [18]. A positive perception of the meaning of life can promote nursing students’ understanding of the meaning of death and a healthy awareness and positive acceptance of death. However, the impact of the COVID-19 pandemic extends beyond individual perceptions of the meaning of life and attitudes toward life and death; it also affects the professional identity of nursing students. Therefore, this study proposes the following hypotheses: (1) Following the COVID-19 pandemic, nursing students’ professional identity is increased, and their sense of meaning in life is decreased. (2) The sense of meaning in life among nursing students is associated with their professional identity, and enhancing their sense of meaning in life will positively contribute to improving their professional identity.

## Methods

### Design, sample and data collection

According to a decision of the State Council of the People’s Republic of China [19],from January 8, 2023, the management of COVID-19 was downgraded from Class A to Class B, following the country’s law on the prevention and treatment of infectious diseases. Accordingly, COVID-19 was managed in China with measures similar to those applied against Class B infectious diseases. Quarantine measures against people infected with novel coronavirus were lifted, with no further implementation of contact tracing or designation of high-risk and low-risk areas. In March and April 2023, a convenience sampling method was employed to select 418 nursing students in Hangzhou, Zhejiang Province. All students were clearly explained the purpose of the study and participated voluntarily.

### Ethical considerations

The study was approved by the University’s institutional review board, and participants were informed that they could withdraw from the study at any time during the completion of the questionnaire.(review number: 2,022,045).

### Instruments

#### The general information questionnaire

A cross-sectional study was conducted using a convenience sampling method. All participants were asked to complete a questionnaire, which consisted of three parts. The first part collected basic information about the students, including gender, education, whether they had clinical practice experience, and whether they had COVID-19 infection.

#### The Chinese version of meaning in life questionnaire (C-MLQ)

The second part measured the nursing students’ perceptions of life meaning using the Chinese version of Meaning in Life Questionnaire (C-MLQ). The scale is a validated research instrument that was developed by Steger et al. in 2006 [20] and translated and revised by S. Liu et al. [21]. The scale comprises two dimensions: Presence and Seeking Sense, consisting of 9 items. Each item was rated on a 7-point Likert scale. Scores < 38, 18–51, and > 51 indicate a low, intermediate, and high sense of life meaning, respectively. The presence of meaningful life refers to the degree to which individuals feel that they are living a meaningful life (emphasis on the result); the search for meaningful life refers to the degree to which individuals actively seek meaning (emphasis on the process).This questionnaire has been used in many studies to assess the sense of meaning in life [22–24]. In this study, the Cronbach’s alpha coefficient for this scale was 0.729.

#### The occupational identity scale

In the third part, we used the Occupational Identity Scale to measure nursing students’ identification with their professional roles. The scale, developed by Hao Yufang [25], comprises 17 items representing five dimensions:career self-concept, benefits of staying and risks of leaving, social comparison and reflection, autonomy of career choice, and social persuasion, using a Likert 5-point scale with a total score range of 17 to 85, of which question 12 was reverse scored. Higher scores represent stronger professional identity of nursing students.This version of the scale is well established in the Chinese context and widely adopted to assess nursing students’ sense of professional identity [10, 26, 27]. The Cronbach’s alpha was 0.827 in Hao’s study [25] and 0.967 in the current study.

### Statistical analysis

Data analysis was performed using SPSS 25.0. Categorical variables were expressed as numbers and percentages, and continuous variables were expressed as mean ± standard deviation (SD). Normality was tested using skewness and kurtosis scores, with absolute values ≤ 3 indicating a normal distribution [28]. Independent samples t-test or ANOVA test was used for one-way analysis. Pearson correlation analysis was used to calculate the correlation between professional identity and sense of life meaning. P < 0.05 was considered statistically significant. Participants were divided into three groups (high, intermediate, and low) based on their scores on the sense of meaning in life scale. An independent samples t-test was used to determine the difference in professional identity between the high- and the low-level life meaning group.

## Results

### Common method bias

This study used self-reported data, so there may be common method bias issues. To address possible common method biases in this study, anonymous, forward-backward counting was used during the testing process. The grading methods are controlled. Confirmatory factor analysis was used to test the common method deviation on all self-assessment items. The results showed that the model fit was very poor: χ^2^/df = 14.545, CFI = 0.717, GFI = 0.418, AGFI = 0.317, NFI = 0.703, RMSEA = 0.180, so there is no serious common method bias problem.

### Career identity and sense of life meaning scores (see table 1)

**Table 1:** Total score of professional identity and sense of life meaning and scores of each dimension

Items		Number of items	Score	Average score of items
Professional Identity	Professional self-concept	6	21.55 ± 6.19	3.59 ± 1.03
	Retention Benefits and Exit Risks	4	14.06 ± 4.18	3.51 ± 1.05
	Social Comparison and Reflection	3	11.28 ± 2.78	3.76 ± 0.93
	Autonomy in career choice	2	7.14 ± 1.95	3.57 ± 0.97
	Social Persuasion	2	7.55 ± 2.08	3.78 ± 1.04
	Total Score	17	61.58 ± 16.16	3.62 ± 0.95
Sense of life meaning	Presence	5	25.10± 7.15	5.02 ± 1.43
	Seeking Sense	4	20.19± 5.99	5.05 ± 1.50
	Total Score	9	45.29± 12.65	5.03 ± 1.41

### Correlation between demographic characteristics of nursing students and sense of life meaning

Table 2 shows the general information of the 418 nursing students and compares the sense of meaningful life scores between different sample characteristics, with significant differences observed in gender, education, and clinical practice experience. Male nurses had significantly higher sense of life meaning scores than female nursing students (*p* < 0.001). Undergraduate nursing students scored higher than specialist and postgraduate nursing students, and there was a significant difference in the overall distribution of the three educational levels (*P* < 0.001). Nursing students without clinical practice experience had significantly higher sense of life meaning scores than those with clinical practice experience (*P* = 0.006). However, no significant difference was observed in their COVID-19 infection history (*P* = 0.207).

**Table 2:** Correlation between demographic characteristics of nursing students and sense of life meaning (*n* = 418)

	Number of people	Ratio(%)	Sense of life meaning								
			mean ± sd				*t*/F				*P*
Gender											
Female	299	71.53	42.23 ± 10.87				*t* = 7.713				<0.001
Male	119	28.47	52.97 ± 13.56								
Education											
Specialty	215	51.44	40.25 ± 10.73			F = 44.420				<0.001	
Undergraduate	127	30.38	52.01 ± 12.18								
Graduate Students	76	18.18	48.32 ± 12.40								
Clinical practice Experience											
Yes	284	67.94	44.07 ± 12.12		*t*=−2.785				0.006		
No	134	32.06	47.87 ± 13.38								
Past COVID−19 infection											
Yes	314	75.12	45.74 ± 12.81	*t* = 1.263				0.207			
No	104	24.88	43.93 ± 12.10								

### Relationship between sense of life meaning and professional identity

Pearson correlation analysis (Table 3) showed that professional identity was significantly and positively correlated with the sense of life meaning (r = 0.703, *P* < 0.01), sense of being dimension (r = 0.649, *P* < 0.01), and sense of seeking dimension (r = 0.709, *P* < 0.01). The nursing students were divided into three groups (high, intermediate, and low) based on their scores on the sense of life scale.ANOVA test showed (Table 4) that high sense of life nursing students had higher professional identity than intermediate (*P* < 0.01) and low (*P* < 0.01) sense of life nursing students.

**Table 3:** Correlation between nursing students’ sense of professional identity and sense of life meaning

Variables	Sense of life meaning	Presence	Seeking Sense
Professional Identity	0.703**	0.649**	0.709**

(**: *P*<0.01)

**Table 4:** Comparison of professional identity scores and differences among nursing students with low, medium and high levels of sense of life meaning

Sense of life meaning	Professional Identity		
	mean ± sd	F	*P*
Low level group(*n* = 121)	49.93 ± 15.02	144.107	*P*<0.001
Medium level group(*n* = 161)	58.49 ± 11.14		
High Level Group(*n* = 136)	75.59 ± 11.33		

## Discussion

This study investigated the relationship between the meaning of life and professional identity among nursing students in China after the COVID-19 pandemic. The findings revealed that the nursing students had a moderate level of life meaning. Furthermore, there was a positive correlation between the sense of life meaning, both in terms of presence and seeking, and their professional identity.

The SARS epidemic in 2003 provided the community with a deeper understanding of the nursing profession, nursing staff, and nursing work [29], consistent with the findings of this study. The average score for professional identity among the 418 nursing students was 61.58 ± 16.16, indicating a moderate level, which was similar to the findings of Zhang et al. during the 2021 epidemic (62.02 ± 12.02) [6]. Furthermore, compared to studies conducted before the COVID-19 outbreak, the scores of nursing undergraduates’ professional identity were improved [30, 31]. These results suggest that the professional identity of nursing students was strengthened after the COVID-19 pandemic and that nursing students demonstrated a higher level of professional identity towards their profession. A cross-sectional study [6] found that 90% of nursing students believed that COVID-19 positively impacts the image of nursing. The scores for social persuasion (3.78 ± 1.04) and social comparison and reflection (3.76 ± 0.93) were higher than those of many researchers before the COVID-19 outbreak [30–34], suggesting that after COVID-19, the positive attitude of the public towards nursing work improves the professional identity of nursing students. Nursing students can strengthen their professional beliefs and explore their future development direction in the nursing industry by self-examination and comparison with other professional fields. Undoubtedly, COVID-19 has tremendously impacted medical, economic, and social development. However, our study suggests that changes in societal and public attitudes towards the nursing profession caused by COVID-19 offer more opportunities than risks in improving the professional identity of nursing students. In line with the hypothesis of this study, nursing students’ professional identity improved after the COVID-19 pandemic.

The concept of having a sense of meaning in life refers to an individual’s perception of their own life purpose and significance [4]. Due to the unique nature of their future progression, nursing students must learn to recognize and face life properly. Compared with the study on life sense of nursing students by many researchers before the outbreak of Covid-19 [35–37], the life meaning score of nursing students decreased in this study(45.29 ± 12.65). Moreover, the score for the seeking life meaning dimension was lower than that of the existing life meaning dimension, attributed to increased uncertainty and evolving circumstances brought by COVID-19, which have had a significant impact on nursing students’ academic, life goals, and even their future employment aspirations [38]. Consequently, nursing students may have developed a negative experience towards the purpose and value of life. At the same time, during periods of strict prevention and control, nursing students may struggle to find the meaning of their own life without a clear sense of direction, unable to find and achieve their own life goals and leading to despair and a sense of meaninglessness. There is a rich literature substantiating [39, 40] that having a clear purpose and life meaning during the COVID-19 pandemic brings a sense of engagement and direction to oneself, which is one of the protective factors against the fear of COVID-19. The presence of a sense of meaning in life is one of the most important components of coping with stressors in difficult times [41], and a high sense of life meaning contributes to individual well-being and life satisfaction, low recognition of life meaning can easily lead to the corresponding negative emotions, which is not conducive to mental health [42].

Herein, we found that male nurses scored higher than female nurses in their sense of life, which may be attributed to men having higher emotional regulation and more active coping strategies. In contrast, women may have higher anxiety and depression levels when facing stressful events. These negative emotions may reduce the interest and motivation for self-development, an important component of life meaning, consequently reducing their sense of meaning. Moreover, nursing students without clinical practice experience scored higher than those with experience. Nursing students without clinical practice experience tend to have more idealized views of the nursing profession, while nursing students with clinical practice experience the impact of nursing theory and reality, complex interpersonal relationships, and other situations during practice [37], leading to confusion about their future and make it difficult for them to find clear life meaning.

Next, we found a significant positive correlation between nursing students’ sense of life meaning and professional identity, which indicated the close relationship between nursing students’ professional identity and life meaning. Moreover, the scores of professional identity of nursing students with a sense of life meaning in high, intermediate, and low groups were significantly different (*P*<0.001). As expected, the scores of professional identity in the high meaning of life group were significantly higher than in the intermediate and low meaning of life groups, indicating that the strength of the sense of meaning in life has an important influence on the professional identity of nursing students. The quest for the meaning of life is an important part of positive psychology. Frankl’s existential analysis theory [43] holds that the basic motivation of human beings is to pursue the meaning and goal of life; the sense of meaning in life affects one’s ability to work, create and bear suffering and also affects one’s psychological development. When an individual’s sense of life meaning is high, his actions towards the goal of professional behavior are associated with positive emotions. In pursuing career goals, sub-goals are achieved step by step through the continuous attention of mental representations, and the resulting pride self-strengthens career preparation. Therefore, individuals with a high level of life meaning may have a higher sense of professional identity. On the contrary, a low sense of meaning in life will damage the individual’s ability to make decisions, leading to a lack of interest in the career and planning motivation and even fear of decision-making, affecting the individual’s professional identity. In recent years, following the impact of COVID-19, the professional value of nurses has been widely recognized and praised by society, and this recognition and respect may have enhanced the nursing students’ sense of life meaning. Nursing students think more about the future and regard nursing as worthy of pride and respect. Professional dignity is enhanced, and their professional identity is further strengthened.

There is an increasing consensus [39, 44] that emergencies such as COVID-19 can affect people’s lives, but training people to actively search for meaning and purpose in life can help them make sense of life events and integrate various experiences with job responsibilities. Those with a strong sense of life meaning are more likely to experience a sense of transcendence, which motivates them to pay attention to others and engage in altruistic behavior [45]. Therefore, cultivating people’s awareness of the meaning and purpose of life may help to improve individuals’ quality of life and enhance their willingness to participate in social services. However, the traditional educational system in China has predominantly focused on exam-oriented education. At present, the exploration of the meaning of life education is still in its preliminary stages, which does not fundamentally carry out life education for nursing students and does not pay attention to the needs of nursing students for life education [46]. In China, some undergraduate colleges only take life education courses as elective courses, and even some junior colleges do not set up special life courses, which leads to a low sense of meaning in the life of junior college nursing students. At the same time, the practical activities of undergraduate colleges are relatively rich, including voluntary activities of organ donation and hospice care, which will promote life thinking by nursing students. In this study, the score of life meaning of undergraduate nursing students (52.01 ± 12.18) was significantly higher than that of junior college nursing students (40.25 ± 10.73). Therefore, the authorities should urge each nursing school to establish a relevant curriculum to improve life education. At the same time, colleges and universities should increase the social practice activities of nursing students, cultivate good psychological quality and humanistic quality of nursing students through multi-channel and deep life education, improve professional identity, and form a professional nursing team that constantly meets the needs of social development.

Our study also has some limitations that should be acknowledged. Firstly, the sample of this study was drawn from nursing students in a specific region, and the generalizability of the findings is limited. A larger sample size from different regions could be considered in future studies to better generalize the findings. Secondly, this study used a cross-sectional survey design, which could not determine causality and whether there were moderating and mediating factors for this relationship. Finally, this study only considered the influence of life meaning on professional identity, and future research can combine other variables to further study the influencing factors and mechanisms of the professional identity of nursing students.

## Conclusion

Given the impact of COVID-19, the professional identity of nursing students in China has improved, although their sense of life meaning has decreased. Nursing students with a relatively high sense of life meaning show stronger professional identity than those with a relatively low sense of meaning. Accordingly, helping nursing students to explore and think about life’s meaning can help them form a strong professional identity.

## Data Availability

Data and materials will be made available on request.
